# Association of Zinc with Anemia

**DOI:** 10.3390/nu14224918

**Published:** 2022-11-20

**Authors:** Sen-Shyong Jeng, Yen-Hua Chen

**Affiliations:** 1 Department of Food Science, College of Life Sciences, National Taiwan Ocean University, Keelung 20224, Taiwan; 2 Institute of Food Safety and Risk Management, College of Life Sciences, National Taiwan Ocean University, Keelung 20224, Taiwan

**Keywords:** zinc, anemia, chronic kidney disease (CKD), trace elements, red blood cells (RBCs)

## Abstract

Zinc is an essential trace element, and anemia is the most common blood disorder. The association of zinc with anemia may be divided into three major forms: (1) zinc deficiency contributing to anemia, (2) excess intake of zinc leading to anemia, and (3) anemia leading to abnormal blood–zinc levels in the body. In most cases, zinc deficiency coexists with iron deficiency, especially in pregnant women and preschool-age children. To a lesser extent, zinc deficiency may cooperate with other factors to lead to anemia. It seems that zinc deficiency alone does not result in anemia and that it may need to cooperate with other factors to lead to anemia. Excess intake of zinc is rare. However, excess intake of zinc interferes with the uptake of copper and results in copper deficiency that leads to anemia. Animal model studies indicate that in anemia, zinc is redistributed from plasma and bones to the bone marrow to produce new red blood cells. Inadequate zinc status (zinc deficiency or excess) could have effects on anemia; at the same time, anemia could render abnormal zinc status in the body. In handling anemia, zinc status needs to be observed carefully, and supplementation with zinc may have preventive and curative effects.

## 1. Introduction

Zinc is one of the most important trace elements in organisms and has three major biological roles: catalyst, structural component, and regulatory ion. In living organisms, zinc is the second most abundant trace element after iron. The average amount of zinc in the adult human body is approximately 1.4–2.3 g. Zinc appears to be a multipurpose element necessary for biological processes, and public health. There have been many reviews on its biochemistry, physiology, pathology and important roles in biology [1,2,3,4,5,6,7,8,9,10]. The association of zinc and zinc deficiency with various diseases has also been reviewed [11,12,13,14,15,16,17,18,19], but the association of zinc and anemia has not been widely reported. Zinc deficiency and excess could contribute to anemia, and abnormal zinc levels (lower plasma zinc and higher erythrocyte zinc) in the blood could be the consequence of anemia. In this review, the effects of zinc deficiency and excess on anemia will be discussed, followed by a description of how anemia could lead to abnormal blood–zinc status in the body, which has been found only recently.

## 2. Anemia

Anemia is a decrease in the total number of red blood cells (RBCs) or the amount of hemoglobin in the blood. Anemia can cause reduced work productivity, poor pregnancy outcomes, increased maternal and perinatal mortality and morbidity, cognitive decline, dementia, and poor educational achievement [20,21]. Anemia is the most common blood disorder. It was estimated that in 2013, anemia affected 27 percent of the world’s population, or 1.93 billion people [22]. The global anemia prevalence accounted for 8.8% of the world’s years lived with disability in 2010 [23]. Anemia results from numerous causes, such as poor nutrition (iron deficiency, vitamin B12 deficiency, folate deficiency, and others) and poor health (bone marrow failure, chronic disease, or thalassemia) [24]. In most cases, iron deficiency is the predominant cause of anemia (>60%). Among people with chronic diseases, people with chronic kidney disease (CKD) have a high prevalence of anemia. CKD-related anemia in individuals under 35 years of age accounts for <10% of the global anemia prevalence, but the proportion increases with increasing age. In the 80+ age group, CKD-related anemia affects approximately 45% of the population, surpassing iron-deficiency anemia by approximately 25% [23]. It seems that at most ages, iron deficiency (nutritional deficiency) is the major cause of anemia, while in older adults, poor health related to CKD is the main cause of anemia.

## 3. Trace Elements Related to Anemia

### 3.1. Iron, Zinc, Copper, Manganese, Molybdenum, and Cobalt Are Related to Anemia

Anemia is pathophysiologically diverse and often multifactorial. Iron is the most abundant trace element, and its deficiency is the leading cause of all types of anemia. Several other trace elements, including zinc, copper, manganese, molybdenum, and cobalt, are also related to anemia [25,26,27,28].

### 3.2. Iron-Deficiency Anemia

Iron-deficiency anemia accounts for >60% of the global anemia prevalence, and certain groups are more vulnerable than others. Among women of reproductive age (aged 15–49 years), the proportion of the global anemia prevalence accounted for by iron-deficiency anemia ranged from 35% to 71%, and among preschool-aged children (aged younger than 4 years), it ranged from 30% to 58% [23]. Because of blood loss during menstruation in females and the increasing iron needs of infants, women of reproductive age and preschool-aged children in particular suffer from a lack of iron. Several chronic diseases, namely, CKD, chronic heart failure, cancer, and inflammatory bowel disease are also frequently associated with iron-deficiency anemia [21,29].

Approximately 60% of the total body iron is present in the hemoglobin in red blood cells, and 7% is in muscle myoglobin; thus, approximately 67% of the body’s iron occurs in proteins that transport or store oxygen. Approximately 12–13% of body iron is found in iron-containing enzymes. Iron is essential for the oxygen transport system and various cellular mechanisms [21,25]. Iron-deficiency anemia can be divided into two main forms: absolute and functional. Absolute iron-deficiency anemia arises in instances of increased demand, decreased intake, decreased absorption, and chronic blood loss. Functional iron-deficiency anemia is a disorder in which the total body iron level is normal or increased; however, iron is not effectively mobilized from the iron stores to the bone marrow or there is a mismatch between iron demand and supply. Absolute and functional iron deficiencies can coexist [21,29].

## 4. Association of Zinc with Anemia

Compared to the established medical literature on iron deficiency [30], the literature on zinc and anemia is very limited [31]. The association of zinc with anemia may be divided into three major forms: (1) zinc deficiency contributing to anemia, (2) excess intake of zinc leading to anemia, and (3) anemia leading to abnormal blood–zinc levels in the body.

## 5. Zinc Deficiency Contributing to Anemia

Deficiency of zinc is widespread and affects systemic growth; metabolism; development of connective tissue, bone and teeth; immune responses; cytokine production; and endocrine regulation [6], but zinc deficiency has only recently been associated with anemia [31].

### 5.1. Zinc Deficiency Coexists with Iron-Deficiency Anemia

Over the years, many reports have indicated that zinc deficiency is significantly associated with iron-deficiency anemia, especially in women of reproductive age and preschool-aged children.

#### 5.1.1. In Women of Reproductive Age

In pregnant Japanese women, zinc status can account for hematological abnormalities to some extent [32]. In a study of 1185 Chinese pregnant women, Ma et al. [33] found that the frequency of iron-deficiency anemia was 51.04% and that the frequencies of iron and zinc deficiency in anemic women were 41.58 and 51.05%, respectively. Among the pregnant women in southern Ethiopia studied by Gibson et al. [34], 29% had iron-deficiency anemia, and 74% had low plasma zinc. A low level of serum zinc was shown to be related to anemia and iron deficiency. In central Sudan, among 200 pregnant women, 52.5% had anemia. Iron-deficiency anemia was prevalent in 6.5% of these women, and 45.0% of them had zinc deficiency [35]. In a study of 351 pregnant women in Nigeria, 63.5% had anemia, and the prevalence rates of iron and zinc deficiency were 64.1% and 49.3%, respectively [36]. In Turkey, serum zinc levels were compared between 30 healthy women and 30 women with iron deficiency (aged between 18 and 60 years). The zinc level in the iron-deficiency anemia group was significantly lower than that in the healthy group [37]. Furthermore, in a case–control study in Iraq, Rasool et al. [38] showed that pregnant women with iron-deficiency anemia had a significantly lower concentration of serum zinc. In Nigeria, iron and zinc deficiency and hematological correlations among anemic pregnant women were studied, and 24/50 (48%) of the pregnant participants were anemic and zinc deficient (44/50, 88%). All of the iron-deficient pregnant women (14/50, 28%) were also zinc deficient, except one [39]. Yokoi et al. [40] indicated that iron-deficient premenopausal women without anemia are likely to be zinc deficient, and zinc pool sizes and iron stores were highly correlated in premenopausal women.

#### 5.1.2. In Preschool-Aged Children

Among children, it was reported that only 35–55% of cases of iron-deficiency anemia are solely due to iron deficiency and that others are associated with changes in the status of multiple trace elements [26]. In Turkey, Ece et al. [41] reported that serum zinc levels were lower in 60 children aged 1–14 years with iron-deficiency anemia than in 64 healthy children. Gürgöze et al. [42] studied the serum zinc level in 52 Turkish children with iron-deficiency anemia and 46 healthy children, all between 1 and 4 years of age. They found that the mean serum zinc concentration was significantly lower in the iron-deficiency anemia group than in the healthy group. In Bulgaria and Ukraine, the serum zinc level in children under 3 years of age with iron-deficiency anemia was lower than that in the controls [26].

#### 5.1.3. In Adults

In addition to pregnant women and preschool-aged children, 43 adults (mean age 35) in Turkey with iron-deficiency anemia also had a high rate of zinc deficiency of 59.5%. Zinc deficiency not only coexists with iron-deficiency anemia but also aggravates the epithelial symptoms (angular cheilosis, glossitis, stomatitis, dysphagia, esophageal web, hair or nail changes, and easy bruising) of iron-deficiency anemia [43]. To determine whether zinc deficiency is associated with iron-deficiency anemia and to assess its effect on symptoms of iron-deficiency anemia, 30 Turkish adults (mean age, 32 years; male/female, 17/13) with iron-deficiency anemia were compared with healthy controls (mean age, 33 years; male/female, 18/12). Patients with iron-deficiency anemia had significantly lower serum zinc levels (43.4 mg/dL) than the control subjects (94.7 mg/dL). Among symptomatic patients, the serum zinc level was significantly lower than that in nonsymptomatic patients. The coexistence of zinc and iron deficiencies can exaggerate the degree and symptoms of iron-deficiency anemia [44].

### 5.2. Zinc and Iron Coexist in Food

Iron and zinc are the two most abundant trace minerals in the human body. Iron and zinc usually have similar levels in foods. Interestingly, most of the above reports of women, children, and adults with zinc deficiency are from developing countries. Zinc deficiency is thought to be uncommon in the United States and other developed countries because the consumption of zinc-rich food is common in these countries [45]. Lim et al. [46] compared foods of animal and plant origin and found that there is a strong association between iron and zinc content in foods. Furthermore, the bioavailability and inhibition of both minerals are affected by the same food components. The cause of iron or zinc deficiency may be inadequate dietary intake of iron or zinc, but the most common causative factor is most likely phytate, which is an inhibitor of iron and zinc absorption. Phytate is present in staple foods such as cereals, corn and rice and has a strong negative effect on iron and zinc absorption from composite meals [10,47,48]. Concurrent iron and zinc deficiencies may be the result of low meat intake and high dietary phytate from staple foods [48].

In human nutrition, iron and zinc are frequently assessed together. Consequently, zinc deficiency and iron deficiency tend to be identified simultaneously [46,49,50]. Graham et al. [50] proposed that a significant proportion of iron-deficiency anemia in humans might be due to zinc deficiency. It was suggested that serum zinc levels should be evaluated in iron-deficiency anemia patients and that combined iron and zinc supplementation should be considered if necessary [33,39,43,44].

### 5.3. In Anemia, Zinc Deficiency May Not Coexist with Iron Deficiency; However, It May Cooperate with Other Factors to Lead to Anemia

Although a significant proportion of zinc deficiency coexists with iron-deficiency anemia in humans, several studies indicate that zinc deficiency may not coexist with iron deficiency in anemia. Cole et al. [45] reported that the prevalence of anemia among low-income minority children from Atlanta, Georgia, USA, was 13.2% (total children surveyed, *n* = 280). Anemic children had a significantly lower mean serum zinc concentration than that of nonanemic children (*p* = 0.004). However, mean serum zinc concentrations were not significantly different between children with low iron stores (based on the ferritin concentration) and those with adequate iron stores (*p* = 0.29).

Greffeuille et al. [31] surveyed zinc status and anemia in 18,658 preschool-aged children in 13 countries and 22,633 nonpregnant women of reproductive age in 12 countries. They found that zinc deficiency was associated with a high prevalence of anemia in preschool-aged children and women of reproductive age in 4 and 5 countries, respectively. The association between zinc and hemoglobin concentrations appears to be independent of iron status in some countries.

Houghton and coworkers [51] studied the relations of iron, zinc, selenium, and vitamin D status with hemoglobin and anemia in 503 children in New Zealand (aged 5–15 years) and found that 4.6% of the children were anemic, 3.2% had depleted iron stores, and none had iron-deficiency anemia. However, the low serum zinc frequency was 14.1%, and zinc deficiency was found to be a risk factor for anemia but did not coexist with iron deficiency. The reason that zinc deficiency did not coexist with iron deficiency in the New Zealand study was suspected to be because in New Zealand, breakfast cereals are often fortified with iron but not zinc. It was also found that selenium might have an indirect effect on anemia.

In a 2016 national micronutrient survey in Cambodia [52], anemia was found in 53% of the 792 Cambodian children studied. In anemic children, the prevalence of zinc deficiency was 39.6%, while that in nonanemic children was 24.8%. Iron deficiency in the anemic and nonanemic children was 10.5% and 8.5%, respectively. Anemia was associated with zinc deficiency but not iron deficiency. However, hookworm infection and hemoglobinopathy were significantly associated with anemia.

### 5.4. Zinc Is Essential for Erythropoiesis

In mammals, erythropoiesis takes place in the bone marrow. The process originates from a multipotent hematopoietic stem cell and terminates in a mature, enucleated erythrocyte [53]. Erythropoietin (EPO) is a humoral cytokine that targets erythroid cells and their progenitors [54]. Erythropoiesis in mammals is regulated by the hormone EPO [55,56].

Several studies using animal models indicate that zinc is essential for erythropoiesis. Huber and Cousins [57] studied the redistribution of body zinc in rat bone marrow after induced erythropoiesis and found that zinc is necessary to support the expansion of the erythrocytic compartment. In pregnant rat dams, erythrocyte production was suppressed in those that were zinc deprived [58]. In another report, young adult mice were fed a zinc-deficient diet for 34 days, and the number of erythroid lineage cells was found to decline by as much as 60%. The results indicated that erythropoiesis was disrupted by zinc deficiency [59]. In addition, King et al. [60] studied the impact of chronic zinc deficiency on erythropoiesis in mice and found that at day 50, the erythroid compartment was reduced by 35%. Konomi et al. [61] suggested that zinc deficiency is associated with depressed hematopoiesis because when rats were fed zinc-deficient diets for 4 weeks, their plasma EPO concentration decreased. Recently, in a series of studies, Jeng and coworkers found that zinc supplementation in vivo stimulates erythropoiesis in rats with phenylhydrazine-induced anemia and those with 5/6 nephrectomy-induced anemia; in vitro, it induces red blood cell formation in the bone marrow [62,63,64]. Evidently, zinc is highly involved in new red blood cell formation.

### 5.5. Zinc Deficiency Alone Does Not Cause Anemia in Rats

Whether zinc deficiency directly causes anemia has been studied in several animal models. Paterson and Bettger [65] studied the effects of zinc deficiency on the hematological profile of male rats (they fed male Wistar rats a diet containing less than 1 ppm zinc for three weeks, and the control rats were fed a diet supplemented with 100 ppm zinc). It was found that zinc depletion resulted in elevated numbers of erythrocytes and hematocrit levels. In addition, the depletion of zinc did not result in a significant change in the erythrocyte age distribution. When 40 male Sprague–Dawley rats were fed an iron-deficient diet (30 mg zinc/kg, no supplemental iron), zinc-deficient diet (4.5 mg zinc and 35 mg iron/kg), or iron/zinc-deficient diet (4.5 mg zinc/kg, no supplemental iron) for 4 weeks, both the iron-deficient and iron/zinc-deficient groups had a significantly lower hematocrit; in contrast, the zinc-deficient group had higher hematocrit levels [61]. Someya et al. [66] fed male rats a zinc-deficient diet (0.7 mg zinc/kg diet) and compared them with those fed a control diet (34.8 mg zinc/kg diet) for 4 weeks, finding that there were no significant differences in the number of red blood cells, hematocrit or hemoglobin concentration between these two groups. The rat model indicates that iron deficiency, or iron/zinc deficiency results in anemia. Zinc deficiency alone does not cause anemia in rats, at least not within a short period, e.g., 4 weeks. 

### 5.6. Zinc Deficiency May Need to Cooperate with Other Factors to Lead to Anemia

Multiple factors can contribute to the occurrence of anemia [31]. The most prevalent risk factors associated with anemia in low- and middle-income countries seem to be nutritional deficiencies, infection/inflammation, and genetic hemoglobin disorders [67]. Based on the analysis of the above data, the following summary statements can be made: 1. Clinical investigation shows that (a) zinc deficiency is highly associated with anemia, and a significant proportion of zinc deficiency coexists with iron deficiency; (b) to a lesser extent, zinc deficiency may cooperate with other factors to lead to anemia; and (c) no reports in humans recognize zinc as a primary factor for anemia. 2. Animal model studies show that (a) zinc is essential for erythropoiesis; (b) in rats, iron deficiency or iron and zinc deficiency results in anemia, but not zinc deficiency alone. At present, there are no data showing a causal relationship between zinc deficiency and anemia. Here, we present the hypothesis that zinc deficiency alone does not result in anemia and that it may need to coexist with other factors to lead to anemia. In humans, the major cooperative factor may be iron deficiency, and the minor cooperative factors may be other nutritional deficiencies, such as folate, vitamin B_12_, vitamin B_6_, riboflavin, and vitamin A, or infection/inflammation [24,67]. The hypothesis we suggested warrants further examination.

### 5.7. Summary of the Association of Zinc Deficiency with Anemia

Deficiency of zinc is widespread; it not only affects the progression of many diseases but also contributes to anemia. The association of zinc deficiency with anemia is summarized in Table 1.

## 6. Excess Intake of Zinc Leads to Anemia

### 6.1. Types of Excess Intake

Zinc is essential for humans, and the recommended dietary allowances for zinc are 11 and 8 mg/day for men and women, respectively [68]. In contrast to zinc deficiency, which is widespread, excess intake of zinc is rare. However, there are case reports on anemia induced by excessive and prolonged intake of oral zinc. Excess intake of zinc may come from overuse of zinc supplements for a long period (e.g., several months or longer) [69,70,71,72,73,74,75,76,77,78,79], high-dose one-time zinc administration for Wilson’s disease [80], use of denture cream (some have zinc concentrations ranging from approximately 17,000 to 34,000 μg/g) [81,82,83], or ingestion of coins (mostly by individuals with mental illness; coins are composed of 98% zinc and 2% copper) [70,84].

### 6.2. High Zinc Levels Induce Copper Deficiency

Zinc is relatively harmless, and many of its toxic effects are due to copper deficiency. There have been several reviews on copper deficiency induced by excess intake of zinc, resulting in anemia and other types of cytopenia [14,85,86]. The syndrome is characterized by anemia, granulocytopenia, and bone marrow findings of vacuolated precursors and ringed sideroblasts. Serum analysis reveals increased zinc levels, decreased copper levels, and a decrease in ceruloplasmin [72].

The mechanism by which high zinc levels induce copper deficiency is based on the assumption that zinc and copper have similar chemical properties and will compete to bind metallothionein. It is suggested that high zinc induces metallothionein formation and that copper is preferentially bound by metallothionein. The transfer of copper bound to metallothionein across the basolateral membrane is blocked, and the copper is subsequently excreted. This process results in copper deficiency [86,87,88,89,90]. However, several studies have shown that another mechanism may be involved. Reeves et al. [91] found that when rats were fed high-zinc diets for two weeks, the metallothionein concentration in intestinal epithelial cells increased, but the copper concentration decreased. This result suggested that intestinal metallothionein induced by high zinc did not bind to copper. Rather than copper being bound by the high zinc-induced mucosal metallothionein, it is more likely that the high zinc in the intestinal lumen directly interfered with the transport of copper across the mucosal cells [91,92,93].

### 6.3. High Zinc Levels May Induce Iron Deficiency

Some reports have shown that excess zinc not only induces copper deficiency but also induce iron deficiency. To study the effects of excess zinc on anemia, Hachisuka and coworkers [94] recently fed a diet with excess zinc to Sprague–Dawley rats for 6 weeks and compared those animals with rats fed a standard diet. In the zinc excess group, microcytic hypochromic anemia was found with a significant increase in serum zinc and a decrease in serum copper and iron levels. The results showed that excess zinc resulted in not only copper deficiency but also iron deficiency. Previously, Yanagisawa et al. [95] fed rats a standard or high-zinc diet (2000 mg/kg) for 20 weeks and showed that the high-zinc diet resulted in a significant decrease in hemoglobin and hematocrit levels. Microcytic hypochromic anemia characterized by iron deficiency was also found. Thus, a high-zinc diet (2000 mg/kg) for 20 weeks may cause iron-deficiency anemia in rats.

### 6.4. Mechanism of Anemia Caused by Copper Deficiency

The exact mechanism of anemia caused by copper deficiency is not clear; however, several reports have shown that copper is essential for normal hematopoiesis. Peled et al. [96] found that under reduced copper conditions, hematopoietic stem and progenitor cell differentiation was delayed, resulting in extended active cell proliferation. However, under elevated copper conditions, differentiation was accelerated, resulting in a shorter phase of cell proliferation. The role of copper in heme synthesis has been well reviewed, and the lack of copper may result in ineffective erythropoiesis [97]. In mammals, four cuproenzymes are referred to as multicopper oxidases, which have iron oxidase activity (ferroxidase) [98]. The mammalian multicopper ferroxidases include ceruloplasmin, hephaestin and zyklopens that are essential for body iron homeostasis. These enzymes function to ensure the efficient oxidation of iron so that it can be effectively released from tissues to the iron carrier protein transferrin in the blood [99]. Copper deficiency is known to result in the impairment of ferroxidase enzymes, which causes impaired hemoglobin synthesis [97,99].

The syndrome of anemia due to excess zinc-induced copper deficiency could be revealed by simple serum copper-and-zinc-level tests. Discontinuation of zinc supplements and oral copper administration can reverse the symptoms [69,72,79,97,100].

### 6.5. Summary of Excess Intake of Zinc Leading to Anemia

Excess intake of zinc is rare. However, there are case reports on anemia induced by long-term, high-dose intake of oral zinc, as shown in Table 2. Excess intake of zinc interferes with the uptake of copper and results in copper deficiency, which leads to anemia.

## 7. Anemia Leads to Abnormal Blood–Zinc Levels in the Body

### 7.1. High Prevalence of Anemia and Abnormal Blood–Zinc Levels in CKD Patients

Anemia of chronic disease is the second most prevalent type after anemia caused by iron deficiency [101]. Among people with chronic diseases, people with CKD have a high prevalence of anemia [23]. It has been shown that 64% and 78% of individuals who suffer from CKD have low serum or plasma zinc levels, respectively [102,103]. As early as 1950, the fact that most CKD patients had plasma–zinc levels lower than those of healthy control subjects was recognized [104,105]. This observation has continued to be reported [106,107,108,109,110,111] and was reviewed by Mahajan et al. [112] and Kimmel et al. [113]. A systematic review and meta-analysis of research from 1966 to April 2008 on trace elements in hemodialysis patients showed that hemodialysis patients appear to have lower levels of zinc than the general population (reviewed by Tonelli et al. [114]). Another review by Neto et al. [115] showed a similar conclusion that lower zinc levels are observed in end-stage renal disease patients on hemodialysis.

Hemodialysis is the most common method of removing uremic toxins from patients with end-stage renal disease. Although the semipermeable membrane may remove uremic toxins from the blood, it may also deplete trace elements, such as zinc. During the hemodialysis process, an abundance of zinc may be excreted. The dialysis process has been speculated to be one of the reasons why CKD patients have lower plasma zinc levels [114]. Other suggested causes of the low plasma zinc levels in CKD patients are dietary restrictions for CKD patients limiting the available zinc in foods and decreased dietary uptake of zinc by the gastrointestinal tract of the patient (reviewed by Neto et al. [115]).

Although only 1% of the total body zinc is present in circulating blood, the plasma–zinc level is the most commonly used index for evaluating individuals and populations at risk of zinc deficiency [116,117]. Because most patients with CKD have lower plasma–zinc levels, it is thought that zinc deficiency might be the cause.

Mahajan et al. [106] showed that although CKD patients have lower plasma zinc levels than healthy people, erythrocyte zinc levels were higher than those of controls regardless of whether they were on maintenance hemodialysis. Higher erythrocyte zinc levels in CKD patients than in healthy people have also been reported in other articles [108,109,110,111,118,119]. Some possible causes for the high erythrocyte zinc levels in CKD patients that have been suggested are abnormal shifts as a result of chronic uremic acidosis [105], ineffective erythropoiesis [106], and an increase in carbonic anhydrase I and II [120,121].

The abnormal blood–zinc levels in CKD patients with anemia have been puzzling to investigators [121,122]. On the one hand, the lower plasma–zinc levels in CKD patients indicate possible zinc deficiency in the patients; on the other hand, higher erythrocyte zinc levels show that the patients may not have a zinc deficiency because the erythrocyte zinc level has been reported to be low in patients with dietary zinc deficiency [123].

### 7.2. Animal Model Studies Indicate That Abnormal Blood–Zinc Levels Might Be the Result of Anemia

Condon and Freeman [105] first proposed that the lower plasma–zinc levels in CKD patients may be derived from the redistribution of zinc in the body of the patients rather than total body zinc deficiency. It was suggested that the movement of zinc from plasma to tissue must occur.

To determine whether the abnormal blood–zinc levels in CKD patients with anemia are caused by zinc deficiency or the redistribution of zinc in the body, Chen et al. [64] recently compared zinc levels in the blood of anemic CKD patients and normal patients and among anemic and normal mice and rats.

Patients on hemodialysis (*n* = 127) had serious kidney disease and anemia. They also had plasma–zinc levels (0.49 ± 0.18 µg/mL plasma) that were significantly lower than those in healthy people (0.98 ± 0.15 µg/mL plasma) (*n* = 21) (*p* < 0.0001). In contrast, the patients on hemodialysis had significantly higher erythrocyte zinc levels (0.72 ± 0.18 μg/10^9^ RBCs) than those of the healthy control group (0.63 ± 0.06 μg/109 RBCs) (*p* = 0.0208).

Five-sixth nephrectomized rats mimic the progression of renal failure after the loss of renal mass in humans and have been a widely used experimental model of CKD [124,125,126]. Chen et al. [64] generated 5/6 nephrectomized rats and fed them a normal diet. Twenty-five days after surgery, the 5/6 nephrectomized rats had significantly lower RBC counts and hematocrit levels than normal rats, and they had severe kidney disease. In the 5/6 nephrectomized anemic rats, the plasma zinc level was significantly lower and the erythrocyte zinc level was significantly higher than those in the normal rats. The changes in the blood–zinc levels in the 5/6 nephrectomized anemic rats compared to normal rats were similar to the changes in the CKD patients compared to healthy people.

Phenylhydrazine administration has been used to generate anemic rats or mice [127]. In this model, phenylhydrazine causes oxidative denaturation or hemolysis of RBCs [128], but the kidneys are not impaired. Chen et al. [64] injected mice with different amounts of phenylhydrazine and fed them a normal diet. After 2 days, the mice became anemic, and the larger the amount of phenylhydrazine injected was, the lower the RBC count in the mice. Furthermore, the lower the RBC count was, the lower the plasma–zinc level and the higher the erythrocyte zinc level in anemic mice. The duration of the mouse experiment involving phenylhydrazine was only 2 days, and the mice were fed a normal diet; therefore, there was no zinc deficiency in the mice.

The assumption that lower plasma zinc levels in CKD patients arise from zinc excretion during dialysis, dietary restrictions, or lower dietary absorption of zinc may be applicable to humans, but it does not explain the lower plasma zinc levels in 5/6 nephrectomized anemic rats and phenylhydrazine-treated anemic mice. During the experiments, the 5/6 nephrectomized anemic rats and phenylhydrazine-treated anemic mice were fed a diet with a normal amount of zinc and did not undergo dialysis. The cause of the plasma zinc decrease in the rats or mice was not zinc deficiency or kidney impairment and was most likely anemia. It is very likely that the abnormal blood–zinc level is the result of anemia.

### 7.3. In Anemia, Zinc Is Redistributed in the Body

To determine whether zinc is redistributed during anemia, Chen et al. [64] measured zinc levels in different tissues of phenylhydrazine-treated rats before and after treatment. Two days after the treatment, the rats were anemic. The zinc levels in soft tissues, including the heart, lungs, thymus, gastrointestinal tract, liver, spleen, kidneys, testes and muscles, remained unchanged before and after the treatment. However, zinc levels in the plasma and bones of phenylhydrazine-treated anemic rats were significantly lower than those before anemia. Several reports [129,130,131,132] indicated that in severely zinc-deficient rats, zinc concentrations were mostly maintained in the muscles, spleen, thymus, liver, kidneys, testes and intestines but were markedly reduced in the bones and plasma. King [133] showed that there is a small pool of zinc with rapid turnover that is located in the bones, liver, and plasma. Chen et al. [64] showed that in anemia, zinc is redistributed. It was further found that the recruited zinc (partly from plasma and mostly from bones) was sent to the bone marrow to produce new red blood cells (reticulocytes). Reticulocytes have a seven-fold higher zinc level than mature erythrocytes. Because zinc is recruited in anemia, the plasma–zinc level decreases; since reticulocytes have much higher zinc levels than mature erythrocytes, in anemia, the erythrocyte zinc level increases.

### 7.4. Lower Plasma Zinc Levels in CKD Patients Could Be a Consequence of Anemia

Based on the above animal model study, the lower plasma–zinc levels in CKD patients could be a consequence of anemia. It is very likely that although zinc deficiency could contribute to anemia, anemia itself could contribute to abnormal blood–zinc levels, as shown in Table 3. In CKD patients, zinc deficiency may contribute to anemia, and anemia might also contribute to abnormal blood–zinc status.

### 7.5. Summary of Anemia Leading to Abnormal Blood—Zinc Levels in the Body

People with CKD have a high prevalence of anemia, and they always have lower plasma–zinc levels but higher erythrocyte zinc levels than those of members of the general population. It was thought that zinc deficiency might be the cause of anemia in CKD patients. However, animal model studies indicate that anemia leads to abnormal blood–zinc status in the body, as shown in Table 3.

## 8. Supplementation of Zinc in Anemia

In many pregnant women and infants suffering from anemia, several reports indicate that zinc supplementation combined with iron therapy can increase hemoglobin levels and improve iron indexes more than iron alone [134,135,136,137,138,139,140,141]. In addition, zinc deficiency is known to be associated with various risk factors for cardiovascular disease. It has been reported that zinc supplementation may help to ameliorate cardiovascular disease risk factors in CKD patients [142].

For CKD patients, in a recent study of 582 hemodialysis patients, 53.6% of CKD patients had a zinc-deficient intake [143]. Many zinc supplementation studies have been performed and reviewed [114,144,145]. Wang et al. [145] performed a meta-analysis after a qualitative synthesis of 15 studies published between 1995 and 2015 from different countries. The mean age of the participants ranged from 13 to 80 years with dialysis for at least 3 months. The elemental zinc doses ranged from 11 to 100 mg per day, and the study duration ranged from 40 to 360 days. The levels of serum zinc and dietary protein intake in the zinc supplementation group were higher than those in the control group after treatment. There was a time–effect but not a dose–effect relationship with zinc supplementation. However, the National Kidney Foundation KDOQI (Kidney Disease Outcome Quality Initiative) clinical practice guideline 2020 suggested that ‘in adults with CKD 1-5D, we suggest to not routinely supplement zinc since there is little evidence that it improves nutritional, inflammatory, or micronutrient status’ [146]. It seems that more studies are needed to recommend how much zinc should be supplemented and how long zinc should be taken.

Concerning the abnormality of zinc levels in CKD patients, several points need to be considered: (1) the anemia in CKD patients may result from zinc deficiency; (2) the lower plasma zinc level in CKD patients may be the consequence of anemia; and (3) the lower plasma zinc may be due to a combination of the above two factors. To normalize the zinc level in CKD patients, it seems that treating anemia and avoiding zinc deficiency should be combined. Fukushima et al. [147] treated CKD patients with recombinant human EPO and supplemented them with oral polaprezinc (beta-alanyl-L-histidinato zinc, 34 mg elemental zinc/day) for 12 months. It was found that the recombinant human EPO needed to maintain a normal hemoglobin level (12 g/dL) significantly decreased 3 months after zinc supplementation, which continued until the end of the trial (12 months). The serum zinc concentration increased significantly from <70 µg/dL to the normal range (80–120 µg/dL) at 1 month and was maintained within the normal range for 1 year. No zinc poisoning from therapy was observed. Kobayashi et al. [148] investigated the effects of zinc supplementation (oral polaprezinc, 34 mg elemental zinc/day for 12 months) on changes in the EPO responsiveness index (weekly erythropoiesis-stimulating agent dose (units)/dry weight (kg)/hemoglobin (g/dL)). The need for an erythropoiesis-stimulating agent (recombinant human EPO) was reduced after 10–12 months, and the serum zinc level increased 3 months after zinc supplementation, which continued until the end of the trial (12 months). Because abnormal zinc levels may also be the consequence of anemia, it may be necessary to examine zinc status in CKD patients and to provide zinc supplementation if necessary.

The use of 5/6 nephrectomized rats as a model for anemia related to CKD showed that zinc supplementation could relieve anemia by inducing new red blood cell formation through another pathway, namely, zinc-induced erythropoiesis [63]. Further research on using zinc supplementation as an inexpensive and reliable treatment to correct anemia in CKD patients and at the same time to normalize zinc levels in blood remains to be performed.

## 9. Conclusions

**Figure 1:**
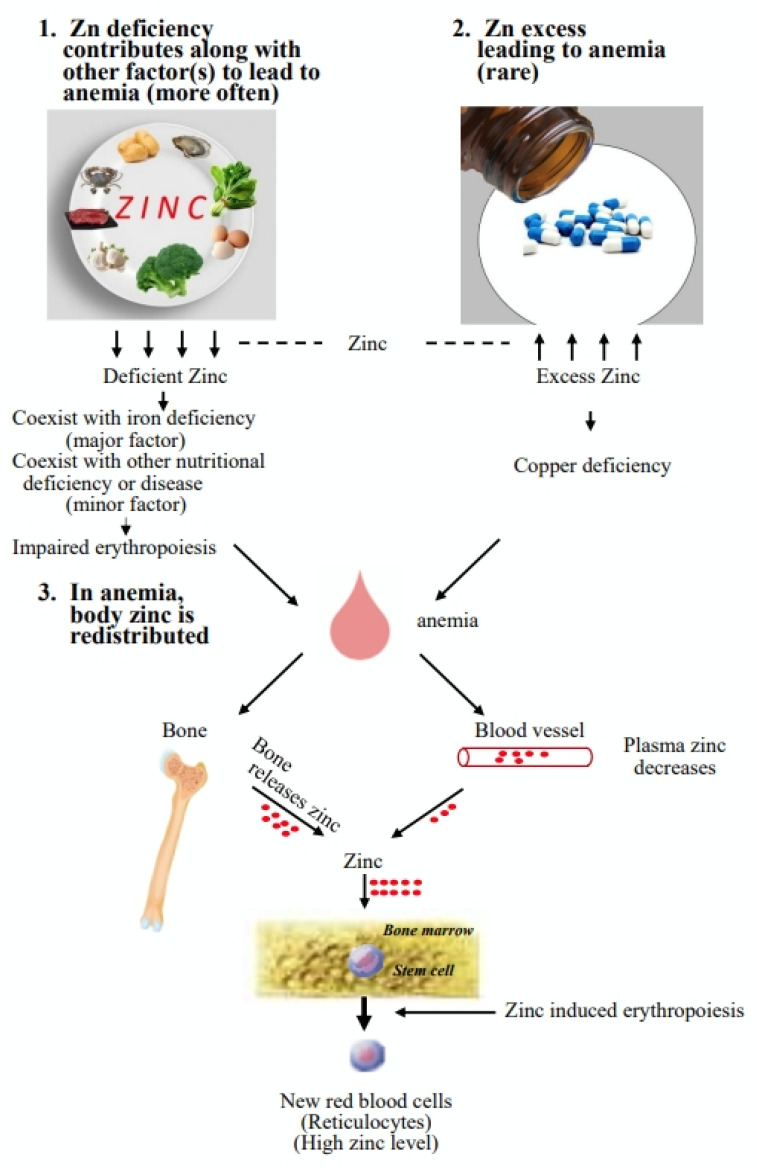
Association of zinc with anemia. Inadequate zinc status (zinc deficiency or zinc excess) could have effects on anemia; at the same time, anemia could render abnormal zinc status in the body.

1. The present review shows that zinc is highly associated with anemia, as shown in Figure 1. In humans at most ages, a significant proportion of cases of zinc deficiency coexist with iron deficiency, which might be the major cause of zinc deficiency contributing to anemia. Serum zinc levels should be evaluated in iron-deficient anemia patients, and combined iron and zinc supplementation should be considered if necessary.
2. For adults or those who have chronic diseases, zinc deficiency might contribute to anemia; at the same time, anemia might render abnormal zinc status. It may be necessary to examine zinc status in CKD patients and to provide zinc supplementation if necessary.
3. The possibility of using zinc compounds as an erythrocyte stimulating agent is high, and further research is needed.
4. The involvement of zinc in anemia is complex, and the interplay of zinc with other factors or diseases in anemia deserves much more attention.

## Figures and Tables

**Table 1:** Zinc deficiency contributing to anemia.

**1.** **In clinical investigation**	**References**
(1) In most cases, zinc deficiency coexists with iron-deficiency anemia	
a. Mostly in women of reproductive age (because they lose blood during menstruation)	[33,34,35,36,37,38,39,40]
b. Mostly in preschool children (because infants need increasing iron and zinc)	[26,41,42]
c. In adults (relatively rare)	[43,44]
d. The reason for the coexistence of zinc deficiency with iron deficiency (The major reason is zinc and iron coexist in food and have same inhibitor, phytate)	[10,45,46]
(2). To a lesser extent, zinc deficiency may cooperate with other factors to lead to anemia	[31,45,51,52]
(3). No reports in humans recognize zinc as a primary factor	[31,45,51]
**2.** **In animal model studies**	
(1). Zinc is essential for erythropoiesis	[57,58,59,60,61,62,63,64]
(2). In rats, iron deficiency or iron+zinc deficiency results in anemia, but not zinc deficiency alone	[61,65,66]
**3.** **A hypothesis is presented: zinc deficiency might need to cooperate with other factor(s) to lead to anemia. In humans the main cooperate factor might be iron deficiency, the minor cooperate factor(s) might be other nutritional deficiency or disease**	

**Table 2:** Excess intake of zinc leads to anemia.

**1.** **Type of excess intake**	**References**
a. Excess and prolonged intake of oral zinc supplement	[69,70,71,72,73,74,75,76,77,78,79]
b. High-dose one-time zinc administration (for Wilson’s disease)	[80]
c. Use of denture cream (containing a high zinc concentration of 17,000–34,000 (µg/g)	[81,82,83]
d. Ingestion of coins (by individual with mental disease)	[70,84]
**2.** **High zinc levels induce copper deficiency**	
(High zinc induces metallothionein, zinc and copper compete to bind metallothionein, and copper is preferentially bound by metallothionein)	[14,72,85,86,87,88,89,90]
(another suggested mechanism is that high zinc in the intestinal lumen directly interfered with the transport of copper across the mucosal cells)	[91,92,93]
*3.* **Mechanism of copper deficiency leads to anemia**	
(Copper is essential for normal hematopoiesis)	[74,96,97]

**Table 3:** Anemia leads to abnormal blood zinc levels in bodies.

**1.** **Blood zinc status in healthy people (controls) and CKD patients**				
(1) Plasma zinc levels	Controls	CKD patients	*p* value	Reference
(µg/mL plasma)	a. 1.07 ± 4 (*n* = 11)	0.82 ± 0.04 (*n* = 10)	<0.01	[105]
	b. 1.016 ± 0.022(*n* = 50)	0.80 ± 0.03 (*n* = 10)	<0.001	[106]
	c. 1.14 ± 0.11 (*n* = 25)	0.95 ± 0.10 (*n* = 26)	<0.001	[109]
	d. 1.105 ± 0.175(*n*= 152)	0.705 ± 0.128 (*n* = 456)	<0.0001	[103]
	e. 0.93 ± 0.12 (*n* = 20)	0.81 ± 0.19 (*n* = 30)	<0.05	[110]
	f. 0.98 ± 0.15 (*n* = 11)	0.49 ± 0.18 (*n* = 127)	<0.0001	[64]
-----Lower plasma zinc levels in CKD patients-----				
(2) Erythrocyte zinc levels				
(µg/g Hb)	a. 44.1 ± 1.1 (*n* = 50)	55.0 ± 2.0 (*n* = 10)	<0.001	[106]
	b. 41.8 ± 63.0 (*n* = 20)	51.3 ± 10.3 (*n* = 30)	<0.05	[110]
(µmoles/L)	c. 148 ± 13 (*n* = 10)	198 ± 8 (*n* = 10)	<0.05	[108]
(mg/L)	d. 12.1 ± 1.2 (*n* = 25)	13.4 ± 3.1 (*n* = 26)	<0.001	[109]
(µg/10^9^ RBC)	e. 0.63 ± 0.06 (*n* = 21)	0.72 ± 0.18 (*n* = 127)	<0.05	[64]
-----Higher erythrocyte zinc levels in CKD patients-----				
**2.** **Blood zinc status in normal (controls) and anemic rats**				
(1) Plasma zinc levels (µg/mL plasma)	Controls	Anemic rats	*p* value	Reference
a. 5/6 nephrectomized anemic rats				
	1.90 ± 0.3 (*n* = 6)	1.40 ± 0.2 (*n* = 6)	<0.05	[64]
b. Phenylhydrazine induced anemic rats				
	1.45 ± 0.06 (*n* = 6)	1.29 ± 0.03(*n* = 6)	<0.001	[64]
-----Lower plasma zinc levels in anemic rats-----				
(2) Erythrocyte zinc levels (µg/10^9^ RBC)				
a. 5/6 nephrectomized anemic rats				
	0.50 ± 0.08 (*n* = 6)	0.90 ± 0.01 (*n* = 6)	<0.001	[64]
b. Phenylhydrazine induced anemic rats				
	0.70 ± 0.21 (*n* = 6)	0.86 ± 0.10 (*n* = 6)	<0.01	[64]
-----Higher erythrocyte zinc levels in anemic rats-----				
**3.** **Animal model studies indicate that in anemia, zinc is redistributed from the plasma (causing the plasma zinc level to decrease) and from bones to the bone marrow to produce new red blood cells (reticulocytes) which have ~7 fold higher erythrocyte zinc levels (causing the erythrocyte zinc level to increase)** [64]				
